# Inhibition of miR-155 reduces impaired autophagy and improves prognosis in an experimental pancreatitis mouse model

**DOI:** 10.1038/s41419-019-1545-x

**Published:** 2019-04-03

**Authors:** Jianhua Wan, Xiaoyu Yang, Yuping Ren, Xueyang Li, Yin Zhu, Ashley N Haddock, Baoan Ji, Liang Xia, Nonghua Lu

**Affiliations:** 1 Department of Gastroenterology, The First Affiliated Hospital of Nanchang University, Nanchang, P. R. China; 2 Department of Cancer Biology, Mayo Clinic, Jacksonville, FL USA; 3 Department of Rheumatology, The First Affiliated Hospital of Nanchang University, Nanchang, P. R. China

## Abstract

Acute pancreatitis (AP) is a common digestive disease characterized by inflammation of the pancreas. MiR-155 plays a role in promoting inflammation and inhibiting the activation of anti-inflammatory pathways. Impaired autophagy could promote zymogen activation, abnormal acinar cell secretion, cell death, and the inflammatory response to aggravate AP. The aim of this study was to ascertain the effect of silencing miR-155 on AP through its effects on inflammation and impaired autophagy in vivo. In this study, AAV(adeno-associated virus)-mediated miR-155 and miR-155 sponge were injected through the tail vein of mice. After 3 weeks, AP was induced by intraperitoneal (IP) injections of cerulein. Pancreatic and pulmonary tissues were analyzed after 24 h. Silencing of miR-155 ameliorated pancreas and lung damage in three AP models of mice by preventing accumulation of autophagosomes that are unable to fuse with lysosomes and decreasing pancreatic inflammation by targeting TAB2. 3-MA could reduce the aberrant accumulation of autophagosomes, which alleviates the pancreas damage that was aggravated by increasing miR-155 levels. These findings demonstrate that the inhibition of miR-155 holds promise for limiting pancreatitis.

## Introduction

Acute pancreatitis (AP) is an inflammatory condition of the pancreas that is characterized by abnormal activation of proteases, parenchymal injury, autodigestion of the pancreas, apoptosis and necrosis of pancreatic acinar cells and an intense inflammatory reaction^1,2^. During the early stage of AP, injury and inflammation in the pancreas can cause systemic inflammatory response syndrome (SIRS), which can cause single or multiple organ failure. SIRS and organ failure are the primary causes of morbidity and mortality in early-stage AP^3^. It is difficult to effectively reduce deterioration in early-stage AP. However, the pathogenesis of organ failure caused by AP has not yet been fully elucidated. Early intervention to reduce systemic or local inflammation could be an effective treatment that improves the prognosis of AP.

MiR-155, a multifunctional miRNA with inflammation-related roles, is regulated by the different types of inflammatory mediators^4^. Bacterial lipopolysaccharide (LPS) and inflammatory mediators, including IFN-β and TNF-α, can induce miR-155 in a human monocytic cell line^5^. In particular, the dysregulation of miR-155 has been strongly implicated in *Helicobacter pylori*-related gastric disease, inflammatory bowel disease, and colorectal cancer through its involvement in molecular changes in important targets and signaling pathways^6^. A previous study showed that miR-155 was upregulated in pancreatitis samples compared with control samples by performing a comprehensive bioinformatics analysis^7^. In the course of AP, the upregulation of miR-155 may promote pancreatic inflammation through its target proteins.

In recent years, autophagy and cathepsin B in lysosomes have become hot topics in pancreatitis, and related studies have revealed some details of the pathogenesis of AP^1,8,9^. Several studies have provided more evidence supporting impaired autophagy and activated inflammation in the origination of AP^1,10^. In the process, impaired autophagic flux causes an accumulation of autolysosomes in acinar cells, which leads to an increase of reactive oxygen species (ROS) due to defective clearance of damaged or depolarized mitochondria, and accumulation of p62-containing protein aggregates. In vitro or vivo, inhibition of autophagy induction by 3-MA reduced the formation of autophagosomes unable to fuse with lysosomes that are observed in cholecystokinin (CCK)-stimulated pancreatic acinar cells or cerulein-induced pancreatitis model^11,12^. Beclin-1 is a central regulator of autophagy whose activity is negatively regulated by the binding with TGF-beta activated kinase 1 (TAK1) /MAP3K7 binding protein 2 (TAB2), which is a miR-155 target protein. In response to pro-autophagic stimuli, TAB2 dissociates from Beclin-1 and establishes a stimulatory interaction with TAK1^13^. Additionally, recent studies suggested that miR-155 promotes autophagy in a variety of diseases^14–16^. We designed this study to investigate the effects of miR-155 on the course of AP by regulating autophagy.

## Materials and methods

### Reagents

Cerulein (Catalog No. C9026), LPS (Catalog No. L4130), L-arginine (Catalog No. A5006), and 3-methyladenine (3-MA) (Catalog No. M9281) were purchased from Sigma-Aldrich (St. Louis, MO, USA). The following primary antibodies used for immunoblotting were purchased from Cell Signaling Technology (Danvers, MA, USA): TAB2 (Catalog No. 3745S), P62 (Catalog No. 5114), and beclin-1 (Catalog No. 3738). The following primary antibodies were used for immunohistochemistry (IHC) or immunofluorescence (IF): microtubule-associated protein 1 light chain 3 (LC3; Catalog No. L8918; Sigma-Aldrich); and green fluorescent protein (GFP) (Catalog No. 6556), TAB2 (Catalog No. ab222214), beclin-1 (Catalog No. ab114071), myeloperoxidase (MPO; Catalog No. ab134132), and caspase-1 (Catalog No. ab1872) (Abcam, Inc., Cambridge, MA, USA). Amylase assay kits (C016-1), lipase assay kits (A054-2), and lactate dehydrogenase (LDH) assay kits (A020-2) were purchased from Nanjing Jiancheng Bioengineering Institute (Jiancheng Biotech, Nanjing, China).

### Mice and treatments

Male BALB/C mice (25 ± 3 g; 6–8 weeks old) were purchased from Hunan SJA Laboratory Animal Co., Ltd. (HSLAC, Hunan, China). All animal protocols were approved by the Institutional Animal Care and Use Committee of The First Affiliated Hospital of Nanchang University and were performed in accordance with the guidelines of the Animal Care and Use Committee. The cerulein pancreatitis model was generated by 10 intraperitoneal (IP) injections of cerulein (100 μg/kg body weight) at 1-h intervals; the cerulein plus LPS model was generated by IP injection of LPS (5 mg/kg) immediately after the 10 IP injections of cerulein (100 μg/kg) at 1-h intervals; and the L-arginine pancreatitis model was created by two IP injections with L-arginine solution (8%, pH = 7.4) at an interval of 1 h and a dose of 4 g/kg^17,18^. 3-MA (20 mg/kg) was administered by IP injection 2 h after the last cerulein injection. The pancreas was harvested 24 h after the last cerulein injection and 72 h after the last L-arginine injection.

### Construction and infection with recombinant AAV-miR-155 and AAV-miR-155 sponge

pAAV9-U6-GFP (adeno-associated virus) vectors carrying miR-155-5p (MIMAT0000165; GATCCGTTAATGCTAATTGTGATAGGGGTTTCAAGAGAACCCCTATCACAATTAGCATTAATTTTTTA), miR-155-5p sponge (MIMAT0000165; ATCGCACCCCTATCATTGTAGCATTAAGGGTCCCACCCCTATCATTGTAGCATTAAGGGTCCCACCCCTATCATTGTAGCATTAAGGGTCCACCCCTATCATTGTAGCATTAAACGCG) or a negative control were generated (Vigene Bioscience, Jinan, China). AAV-miR-155 and AAV-miR-155 sponge were injected via the tail vein in 50 μl PBS per mouse. Mice showed adequate transfection 3 weeks after AAV-9 administration.

### Histological analysis

Pancreas and lung tissue samples were fixed in 10% formalin for 24 h, embedded in paraffin, and sectioned. The sections were processed for hematoxylin and eosin (H&E) staining. Pancreatic histopathology was scored based on Schmidt et al.^19^ on a scale of 0–3 in four items as previously described^11,20^: edema (0, absent; 1, focally increased between lobules; and 2, diffusely increased); inflammatory cell infiltration (0, absent; 1, in ducts or around ductal margins; 2, in the parenchyma of <50% of lobules; and 3, in the parenchyma of >50% of lobules); hemorrhage and fat necrosis (0, absent; 1, 1–2 foci; 2, 3–4 foci; and 3, >5 foci); and acinar necrosis (0, absent; 1, periductal necrosis <5%; 2, focal necrosis 5–20%; and 3, diffuse parenchymal necrosis 20–50%).

### Measurements of amylase, lipase, and LDH activity

Blood was collected by cardiac puncture and centrifuged at 3000 rpm for 10 min to collect serum. Serum amylase and lipase activity (U/L) was measured using a commercially available kit (Jiancheng Biotech, Nanjing, China). LDH activity was determined using LDH detection kits according to the manufacturer’s instructions (Jiancheng Biotech, Nanjing, China).

### Quantitative real-time polymerase chain reaction

Total RNA was extracted from pancreatic tissues using the miRNA kit (Tiangen, China). cDNA was synthesized, and quantitative real-time polymerase chain reaction (PCR) was performed with an miRNA kit (Tiangen, China). Primers for miR-155 were obtained from Tiangen. 5S served as the internal reference.

### Immunofluorescence

Samples were fixed in 4% paraformaldehyde for 1 h, permeabilized in 0.1% Triton X-100 for 30 min, blocked in 10% goat serum and incubated overnight at 4 °C with the primary antibody and then for 1 h at room temperature in the dark with the secondary antibody (Invitrogen). Cell nuclei were stained by the nuclear fluorochrome 4′,6-diamidino-2-phenylindole (DAPI, 1:5000, Invitrogen). The slides were photographed using a fluorescence microscope (Olympus, Tokyo, Japan).

### Electron microscopy, IHC, and western blotting

A detailed description of the materials and methods is provided in our previous study^11^.

### Statistical analysis

The data are expressed as the mean ± SEM. Statistical analyses, including the Mann-Whitney nonparametric *U* test and two-tailed Student’s *t*-test, were performed using SPSS statistical software 20.0 (IBM Corp., Armonk, NY, USA). In all cases, *P* values < 0.05 were considered statistically significant.

## Result

### MicroRNA-155 overexpression in pancreatic tissues mediated by AAV-9 regulates TAB2 expression

**Fig. 1:**
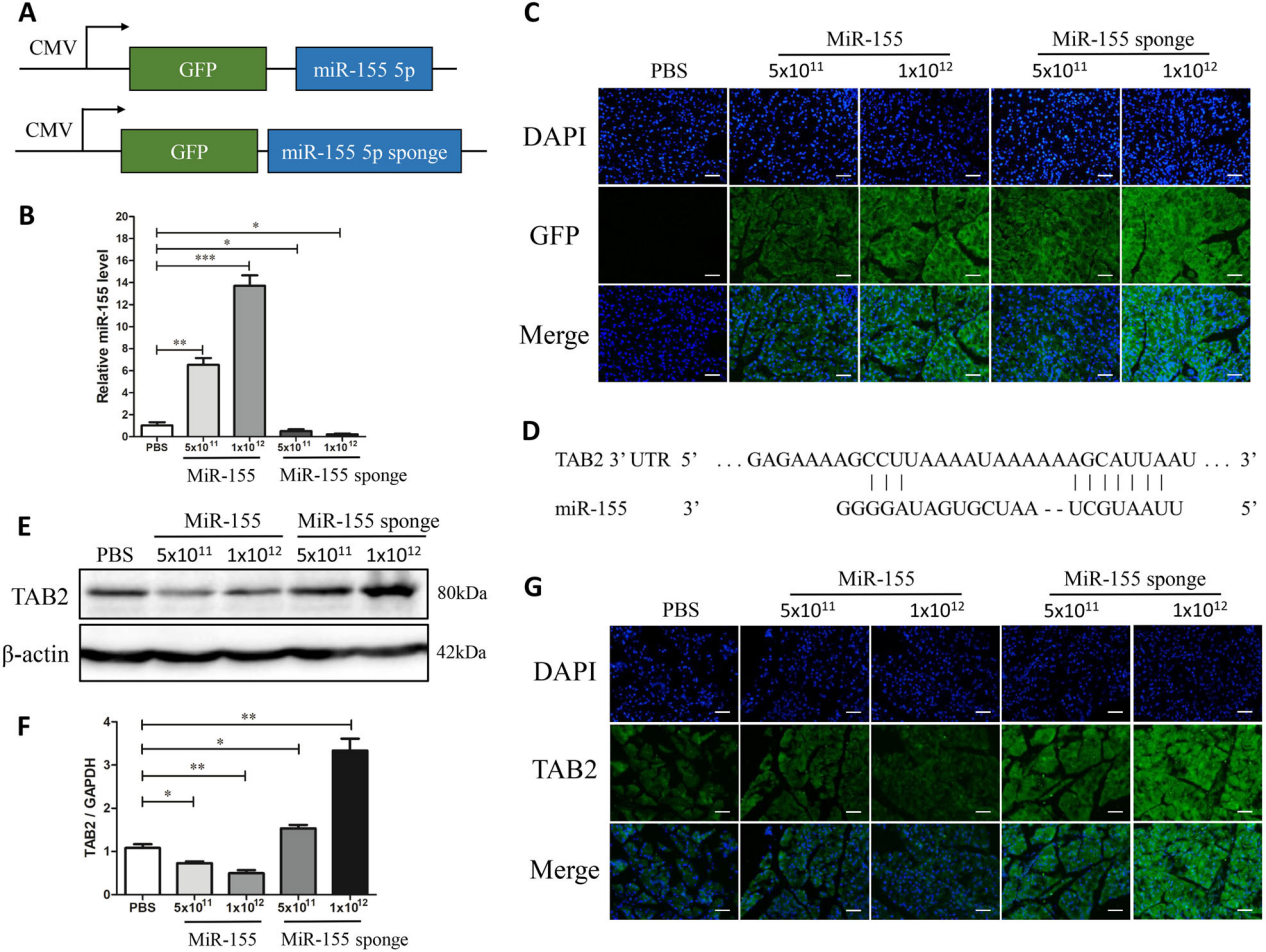
MiR-155 is delivered by AAV-9 to the pancreas. **a** Schematic representation of AAV-9 used in this study with the locations of the miRNA and GFP indicated. **b** Real-time PCR analysis of miR-155 levels after 3 weeks of transfection with AAV-9 harboring miR-155 or miR-155 sponge (*n* = 6 per group, *P < 0.05, **P < 0.01, ***P < 0.001). **c** Fluorescence microscopy showing efficient transduction of pancreas, as indicated by GFP expression, 3 weeks following AAV administration (×200). **d** Sequence alignment of TAB2 with the seed sequence of miR-155 using bioinformatics algorithms. The target prediction program identified a potential miR-155 binding site in TAB2. **e**–**g** Western blot and IF results showing that TAB2 expression is regulated by miR-155 (*n* = 3 per group, *P < 0.05, **P < 0.01)

We injected an AAV-9 vector expressing miR-155 or miR-155 sponge with GFP via the tail vein into BALB/C mice (Fig. 1a), which were sacrificed three weeks later. As illustrated in Fig. 1b, miR-155 expression in pancreatic tissue was elevated in the two miR-155 groups and was significantly lower in the two miR-155 sponge groups compared to the control group. The high AAV-9 titer had a pronounced effect on miR-155 expression in each group, as shown by qPCR and GFP IF (Fig. 1b, c). Based on the miRNA database and previous reports, TAB2 is a predicted miR-155 target. The 3′UTR of TAB2 contains a potential binding site for miR-155 (Fig. 1d). TAB2 expression was detected by western blot and IF after injection of AAV-miR155 or AAV-miR-155 sponge. TAB2 expression levels in pancreatic tissues decreased following AAV-miR-155 sponge injection but increased after AAV-miR-155 injection (Fig. 1e–g). These results indicate that AAV-miR155 and AAV-miR-155 sponge were successfully transfected into pancreatic tissues, where they regulated the expression of TAB2.

### Modulation of miR-155 expression in pancreatic tissues can affect the severity in cerulein-induced AP

**Fig. 2:**
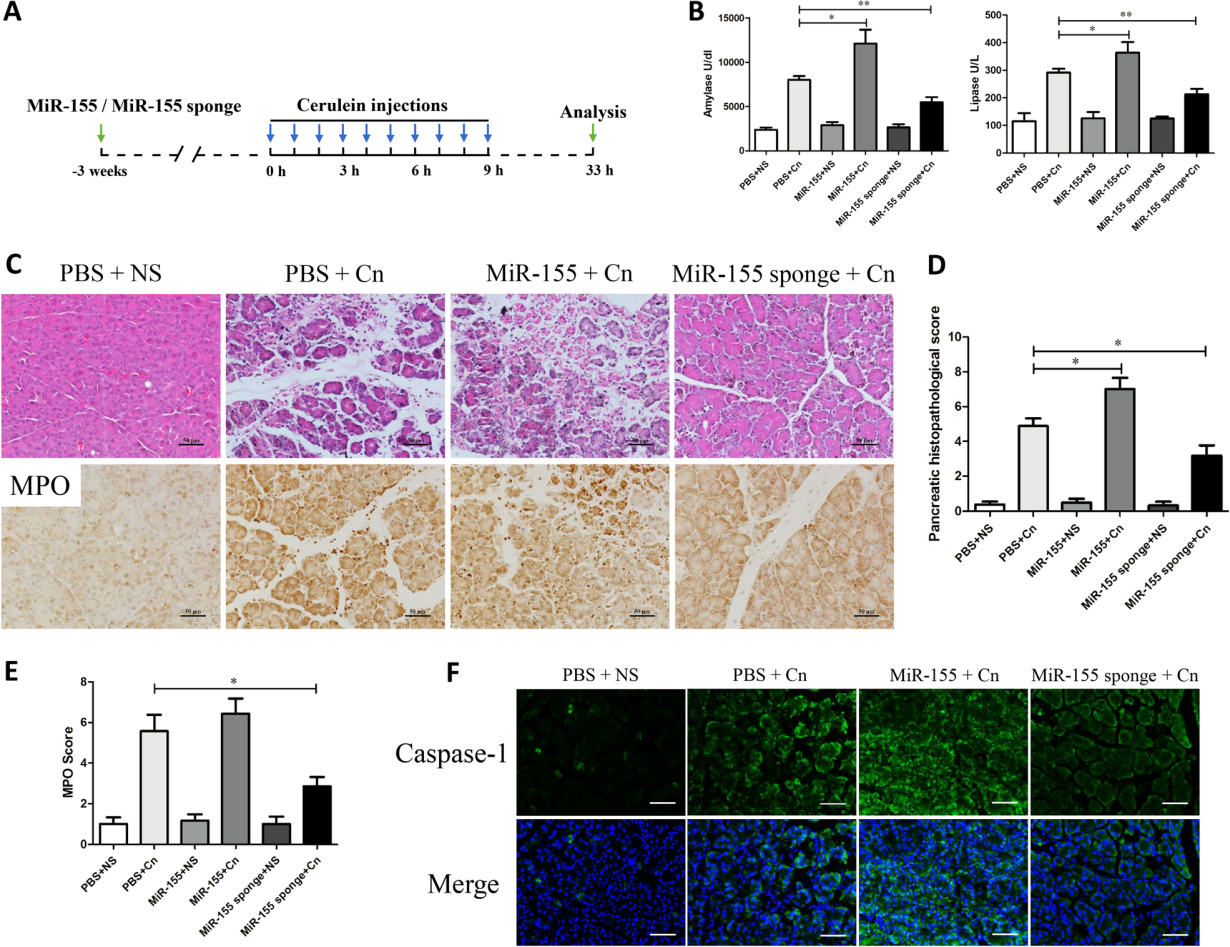
Regulation of miR-155 affects the severity of cerulein-induced pancreatitis in vivo. **a** AP was induced in BALB/c mice with cerulein (100 μg/kg × 10) after 3 weeks of transfection with AAV-9-miR-155 or AAV-9-miR-155 sponge. The mice were sacrificed under anesthesia 24 h after the last injection of cerulein. **b** Serum amylase (U/L) and serum lipase (U/L) activity (*n* = 8 per group, *P < 0.05, **P < 0.01). **c**–**e** H&E staining of pancreas tissue from each group showing different levels of tissue damage, including pancreatic edema, extravascular infiltration and acinar cell necrosis (×200). Immunohistochemical staining and quantification of MPO revealed inflammation in the pancreas (×200) (*n* = 8 per group, *P < 0.05). **f** IF staining for caspase-1 in the pancreas (×200)

To investigate the participation of the miR-155 in cerulein-induced pancreatitis, AP was induced by 10 hourly IP injections of a supramaximal dose of cerulein (100 μg/kg) after three weeks with AVV-miR155 or AAV-miR-155 sponge injection (Fig. 2a). In the three control groups treated with normal saline (NS), the injection of PBS, AAV-miR155 or AAV-miR-155 sponge did not produce any evidence of pancreatitis, as measured by pancreatic edema, inflammatory cell infiltration, pathological changes in acinar cells, and amylase and lipase levels (Fig. 2b, c). Histological examination of the pancreas after cerulein induction showed tissue injury characterized by marked edema, inflammatory cell infiltration and a large number of necrotic acinar cells (Fig. 2c, d and Suppl Fig. 1). In the control groups, cerulein-exposed mice injected with scrambled versions of miR-155 and miR-155 sponge showed no difference from those untreated (Suppl Fig. 1). Pancreatic histopathology score and serum amylase and lipase activity were significantly reduced in the AAV-miR-155 sponge-treated cerulein-exposed mice compared with the untreated cerulein-exposed mice. In contrast, the pathological damage in the pancreas was still very serious in cerulein-exposed mice following treatment with AAV-miR155 (Fig. 2b–d). MPO activity in the pancreas reflected the neutrophil infiltration into the damaged tissue. The number of MPO-positive cells was lower in the AAV-miR-155 sponge-treated cerulein-exposed group than in the untreated cerulein-exposed group (Fig. 2c, e). Caspase-1 was analyzed as an inflammatory response initiator of the inflammatory response, which exhibited lower levels in the miR-155 sponge group than AP without treatment (Fig. 2f). The TUNEL staining analyses showed that miR-155 increased apoptosis, while miR-155 sponge clearly decreased apoptosis in AP (Suppl Fig. 2). Thus, elevated miR-155 may aggravate the severity of cerulein-induced AP, and downregulation of miR-155 may protect against experimental pancreatitis.

### Silencing miR-155 alleviates lung damage in mice with cerulein-induced AP

**Fig. 3:**
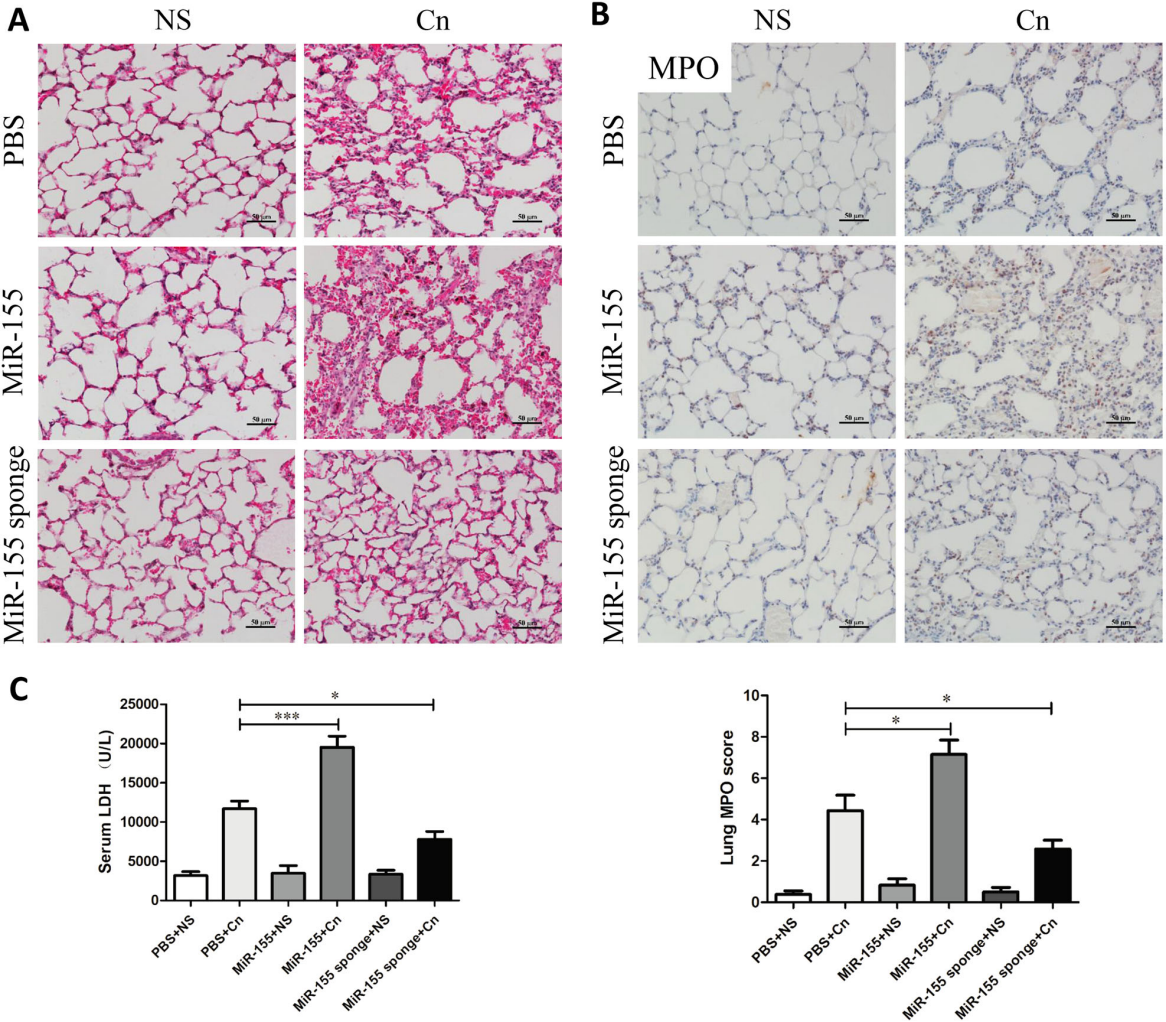
Downregulation of miR-155 alleviates lung damage in mice with AP. **a** H&E staining of lung samples from the six groups of mice revealed different levels of tissue injury, including alveolar thickening and inflammation (×200). **b** Immunohistochemical evaluation of inflammation in mice based on MPO expression levels in the lung (×200). MPO immunohistochemical scores (*n* = 8 per group, *P < 0.05.) **c** Serum LDH (U/L) activity. Each value represents the mean ± standard deviation (*n* = 8 per group, *P < 0.05, ***P < 0.001)

Respiratory failure is one of the most frequent complications of AP and is often related to a high risk of death. The cerulein-only treatment group showed pathological features such as slightly thickening of the alveoli, neutrophil infiltration, and alveolar congestion (Fig. 3a). In miR-155-treated mice, the lungs showed more thickening of the alveoli and neutrophil infiltration. However, exposure of the AAV-miR-155 sponge-treated mice to different doses of cerulein led to a marked reduction in pathological characteristics (Fig. 3a). Similarly, MPO expression levels in the lung were significantly higher in miR-155-treated mice than in those treated with cerulein only. With treatment of AAV-miR-155 sponge, the positive rate of MPO was significantly lower by evaluating lung MPO score in comparison with the untreated group (Fig. 3b). Therefore, silencing miR-155 could effectively reduce neutrophil transmigration into the lungs. Moreover, LDH activity was examined to evaluate necrosis degree of the cells. Mice treated with cerulein showed increased serum LDH activity, and silencing miR-155 significantly reduced LDH activity (Fig. 3c).

### Modulation of miR-155 expression improved TAB2-dependent autophagy by in cerulein-induced AP mice

**Fig. 4:**
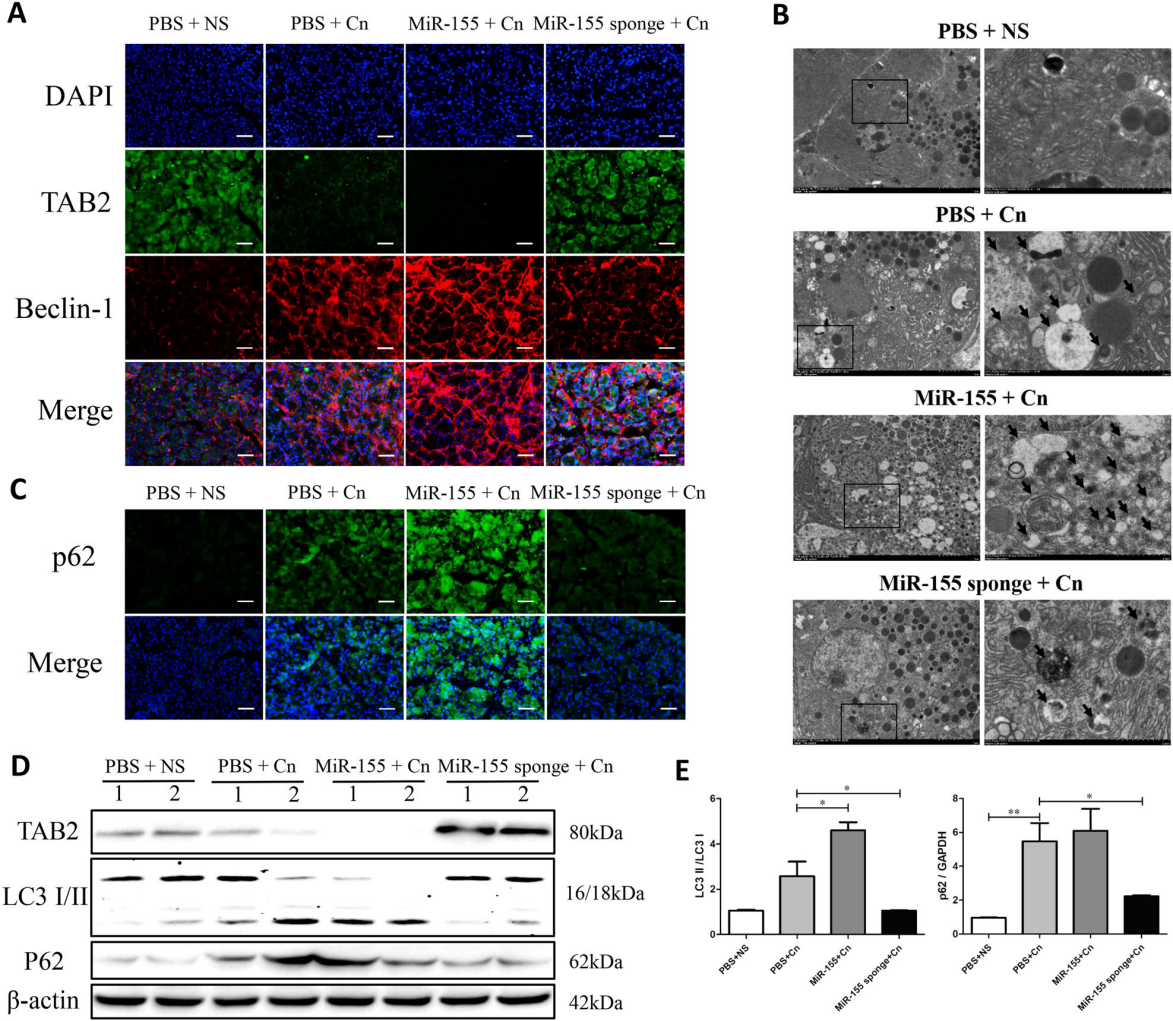
Silencing miR-155 ameliorates TAB2-induced impaired autophagy in mice with AP. **a** IF staining for TAB2 and beclin-1 in the pancreas (×200; green, TAB2; red, beclin-1). **b** Autophagic vacuoles were detected in pancreatic tissue samples by TEM (×10,000 and ×50,000; arrow, autophagic vacuole). **c** IF staining for p62 in the pancreas (×200). **d** Protein expression and analysis of TAB2, p62 and LC3-I/II by western blotting. The gray values were calculated, and data are presented as the mean ± standard deviation (*n* = 6 per group, *P < 0.05, **P < 0.01)

To further explore the mechanisms involving miR-155 that affect pancreatic injury, we investigated whether miR-155 and TAB2 influence autophagy in the AP mouse model. In AP, impaired autophagosome maturation could activate oxidative stress and nuclear factor κB (NF-κB) pathways to aggravate the disease process^10^. In our previous results, inhibition of autophagy initiation by 3-MA could significantly reduce the severity of pancreatitis pathology and improve prognosis^11^. Based on previous studies^13^ reporting that TAB2 interacts with Beclin-1 and inhibits autophagosome formation, we performed a double-staining with anti-TAB2 antibody and anti-beclin-1 to understand if TAB2 may be involved in the regulation of autophagy in our experimental settings. Interestingly, down-regulation of miR-155 expression results in an increase of TAB2 levels associated to a lower expression of beclin-1 (Fig. 4a and Suppl Fig. 3). Downregulation of miR-155 increased TAB2 expression and led to lower beclin-1 levels (Fig. 4a and Suppl Fig. 3). Excessive p62 accumulation and cytoplasmic vacuolation (especially with LC3 II) are markers of impaired autophagic flux^1^. To determine whether autophagy was impaired, the expression levels of the autophagy proteins beclin-1, p62 and LC3-I/II were detected by IF, IHC or western blotting. Impaired autophagy was observed in the course of AP, with elevated levels of beclin-1, p62, LC3-II, and cytoplasmic vacuolation compared with the control (Fig. 4b–e and Suppl Fig. 4). The percentage of autophagic vacuoles per cytoplasmic area was significantly increased in the AAV-miR-155 with cerulein group compared with the cerulein-only group, as determined by transmission electron microscopy (TEM). However, AAV-miR-155 sponge administration significantly decreased the percentage of autophagic vacuoles per cytoplasmic area compared with cerulein alone (Fig. 4b). In addition, miR-155 silencing reduces the accumulation of autophagosomes that are consequence of an impaired autophagic flux, as shown by the decreased levels of LC3II and p62 (Fig. 4c–e). On the other hand, miR-155 overexpression further inhibits the expression of its target protein of TAB2 worsening features of impaired autophagy flux (Fig. 4b–e). Thus, effective reduction of miR-155 could alleviate the severity of AP by decreasing accumulation of undigested autophagosomes in cerulein-induced mice.

### Role of miR-155 on cerulein plus LPS or L-arginine-induced SAP mice

**Fig. 5:**
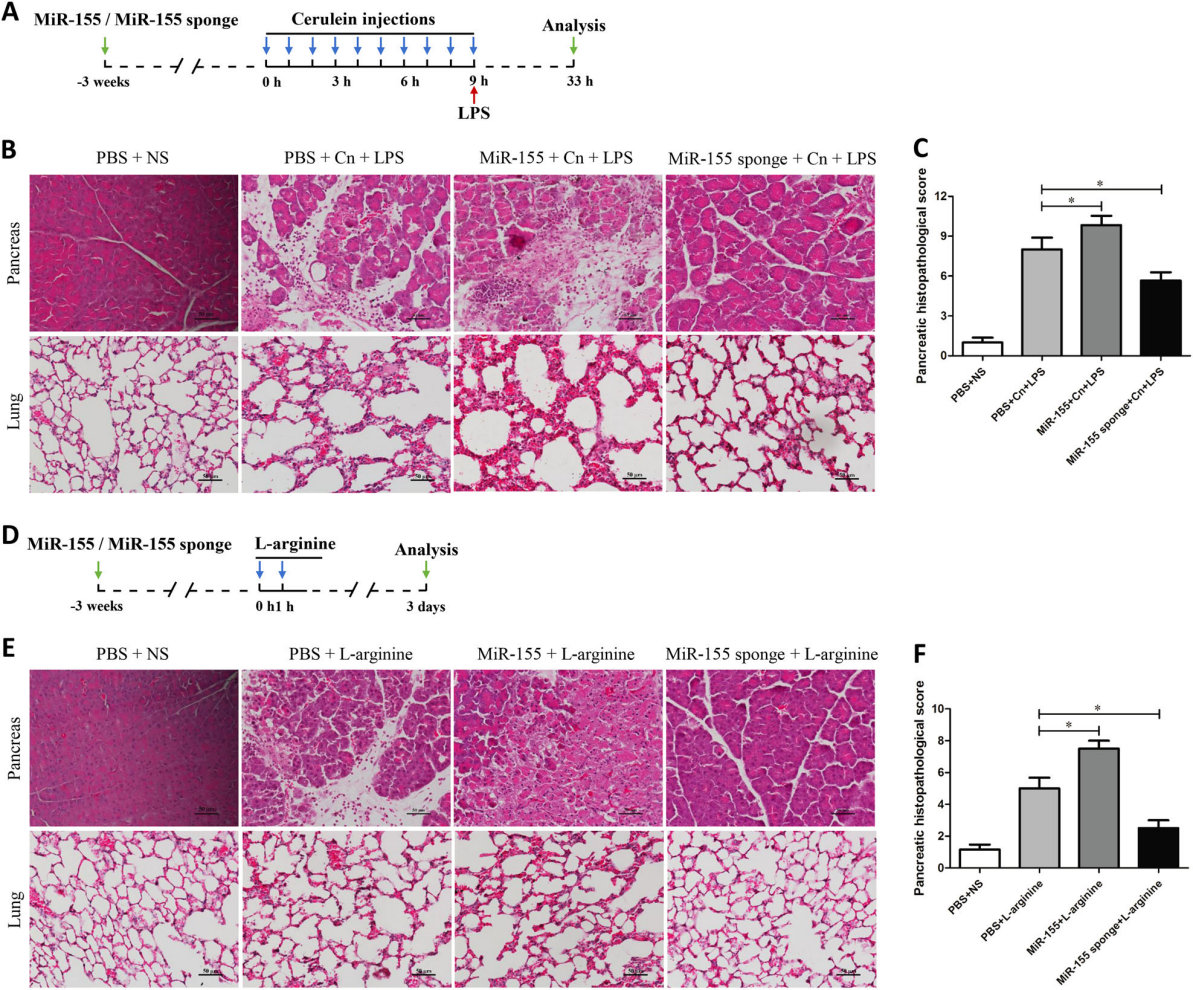
The effects of miR-155 on mice with SAP induced by cerulein plus LPS or L-arginine. **a**, **b** SAP was induced in BALB/c mice by cerulein combined with LPS, and histological analysis (×200) revealed pathological damage in the pancreas and lung. **c** Histopathological score of pancreatitis severity (*n* = 6 per group, *P < 0.05). **d**, **e** SAP was induced in BALB/c mice by L-arginine, and histological analysis (×200) revealed pathological damage in the pancreas and lung. **f** Histopathological score of the pancreas in the L-arginine model. Each value represents the mean ± standard deviation (*n* = 6 per group, *P < 0.05)

A model of SAP (severe acute pancreatitis) induced by cerulein combined with LPS or L-arginine has been characterized in previous studies^11^. In our study, histology of pancreas and lung shows characteristic changes of SAP after treatment with cerulein combined with LPS or L-arginine (Fig. 5a, d). The histopathology alterations cause by cerulein plus LPS in pancreas and lung were greatly improved by miR-155 pretreatment (Fig. 5b). Compared with untreated group, pancreatic histopathological score in the pancreas of miR-155 mice was increased, while it was decreased in the miR-155 sponge group (*P* < 0.05) (Fig. 5c). Similar to the results obtained in the cerulein plus LPS model, treatment with miR-155 sponge reduced pathological damage in pancreas and lung of cerulein plus L-arginine when compared to control treated group (Fig. 5e, f). These results demonstrate that knockdown of miR-155 could alleviate pathological injury of pancreas and lung in other two models of SAP in mice.

### Inhibition of autophagy initiation alleviates severity of AP aggravated by miR-155 overexpression

**Fig. 6:**
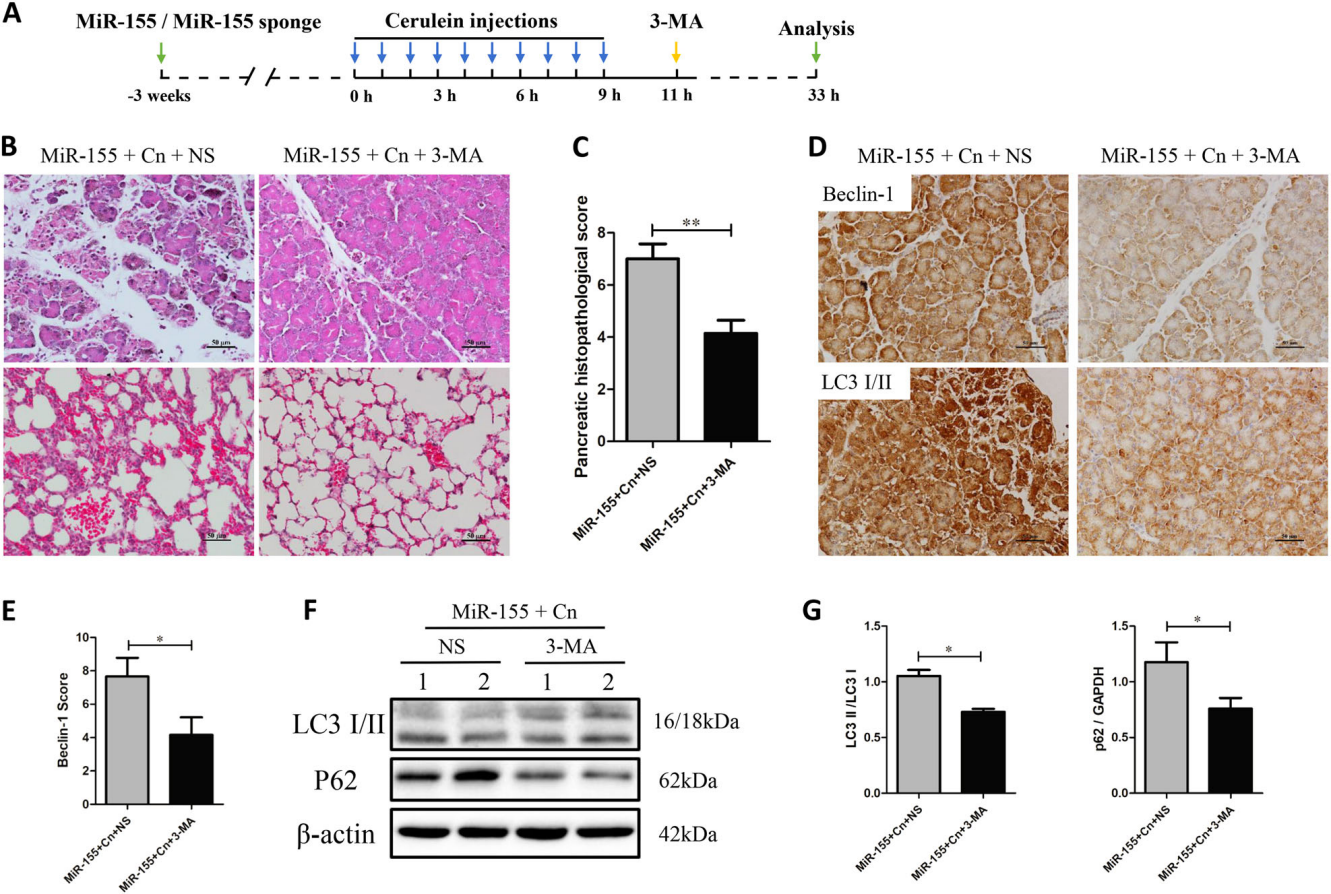
3-MA treatment ameliorates pancreatic damage aggravated by miR-155 upregulation in mice. **a** AP was induced by cerulein as previously described. 3-MA was administered to the AAV-9-miR-155 and untreated groups to treat AP. **b** H&E staining of the pancreas and lung (×200). **c** Pancreatic histopathological score (*n* = 6 per group, **P < 0.01). **d** Immunohistochemical evaluation of beclin-1 and LC3-I/II expression levels in the pancreas (×200). **e** Immunohistochemical scores of beclin-1 (*n* = 6 per group, *P < 0.05). **f**, **g** Protein expression and analysis of p62 and beclin-1 by western blotting (*n* = 6 per group, *P < 0.05)

In order to confirm the role of impaired autophagy flux in AP, we tested the effect of the well-known autophagy inhibitor 3-MA (Fig. 6a)^11^. TOur results show that miR-155 aggravates cerulein-inducing pancreatitis by impairing autophagy flux, which causes more severe inflammation and necrosis. Notably, inhibition of autophagy initiation by 3-MA in mice treated with cerulein plus miR-155 is able to reduce edema, inflammation and necrosis in pancreas and the thickening of the alveoli, neutrophil infiltration and alveolar congestion in lung (Fig. 6b, c). Inhibition of autophagosome formation by 3-MA in cerulein plus miR-155 group was confirmed by the decrease in beclin-1 and LC3 level by IHC and LC3 II and p62 by WB (Fig. 6d–g). 3-MA also reduces the number of LC3 puncta, an indication of reduced accumulation of undigested autophagosomes (Fig. 6d). Altogether, these data indicate that miR-155 may aggravate the severity of AP by impairing autophagic flux, which could be reduced by using an inhibitor of autophagosome formation.

## Discussion

**Fig. 7:**
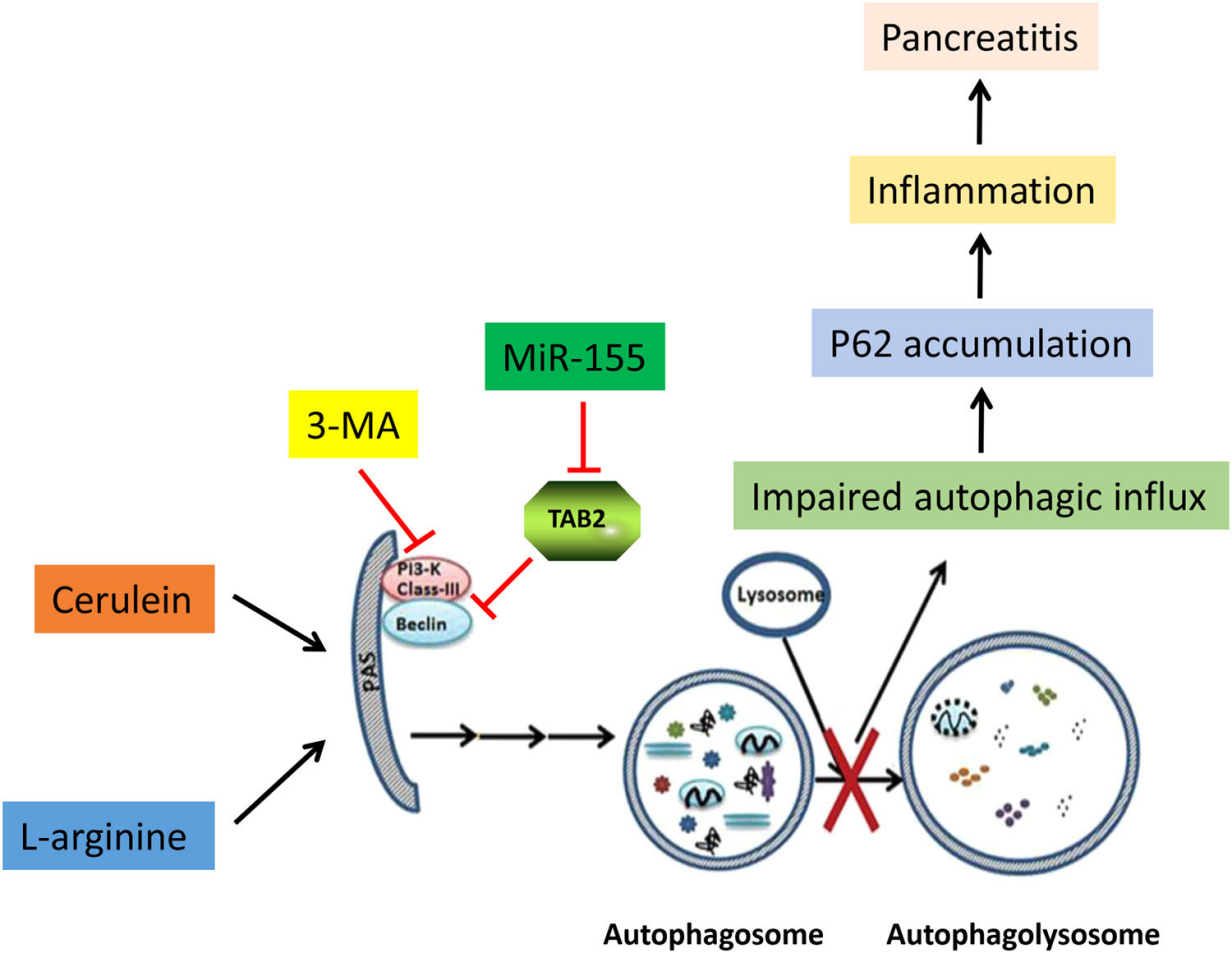
**The mechanism underlying the regulation of miR-155 and autophagy in AP**

In the present study, we identified miR-155 as a regulator of pro-inflammatory mediators that worsens AP in mice. We found that miR-155, by targeting TAB2 gene, promotes the formation of autophagosomes that accumulate because unable to complete maturation in response to AP insult. On the other hand, silence of miR-155 inhibited autophagy initiation and decreased the inflammation of pancreas in AP (Fig. 7). The protective effect of anti-miR-155 was further confirmed in mice with AP.

Autophagy is the primary catabolic process by which cells remove damaged, impaired or unwanted organelles and long-lived proteins and lipids and reclaim the constituents to meet energy and biological demands^21^. Several studies have been conducted on the function and mechanism of autophagy over the past decade, revealing its primary roles in homeostasis, metabolic adaptation, quality control of intracellular organelles, differentiation and development^22,23^. In the process of autophagy, autophagosomes are formed by encapsulation of the degraded material and then transported to the lysosome, leading to the formation of autophagolysosomes, in which organelles undergo renewal by enzymatic hydrolysis, digestion and degradation^24^. Among them, LC3 is an essential protein for autophagosome formation and autophagic vesicles and is a universal marker of autophagic membranes. There are two forms of LC3, types I and II; LC3-II is located on the membrane of intracellular autophagosomes, and its content is proportional to the number of autophagic vacuoles^24^. SQSTM1, also known as p62, is an autophagosome cargo protein that functions in selective autophagy to target and degrade other proteins^25^. P62 is involved in the regulation of a variety of cellular processes, including autophagy and oxidative stress^26^. Under normal physiological conditions, autophagic flux is free of autophagic vesicle accumulation. The accumulation of autophagosomes can be increased by autophagy formation or lysosomal fusion disorders, and the accumulation of autophagolysosomes leads to lysosomal degradation disorders^27^. P62 is specifically degraded by autophagy and remains at low levels under normal physiological conditions. Thus, excessive accumulation of p62 and formation of cellular vacuoles (particularly with LC3-II) are markers of impaired autophagy^1^. The regulation of autophagy involves many signaling pathways and molecular mechanisms that have not been fully elucidated. 3-MA blocks autophagosome formation by inhibiting the activity of class III PI3Ks^24^.

In vitro experiments confirmed that high-dose CCK stimulates pancreatic acinar cells, leading to the formation of a large number of cell vacuoles, the accumulation of p62, an increase in LC3-II and the activation of trypsinogen, suggesting impaired autophagy^12^. An animal model of pancreatitis showed that impaired autophagy was mainly caused by lysosome dysfunction, and autophagy progressed slowly due to the damaged protein degradation function of autophagolysosomes^12^. A large number of vacuoles and the accumulation of trypsinogen in acinar cells can be observed in patients with pancreatitis; the trypsinogen zymogen is activated to induce the development of AP^10,28,29^. Impaired autophagy can promote AP through many mechanisms, such as mitochondrial damage, p62 accumulation, endoplasmic reticulum stress, and ROS release, thereby promoting zymogen activation, abnormal acinar cell secretion, cell death, and the inflammatory response^1,27^. P62 accumulation causes endoplasmic reticulum stress, which in turn causes NF-κB activation and induces inflammation-related protein transcription^30^. In vitro inhibition of autophagy formation by 3-MA reduces the formation of damaged autophagic vesicles caused by CCK stimulation of pancreatic acinar cells^12^. Similarly, our previous study showed that a reduction in the formation of non-functional autophagosomes by 3-MA reduced the severity of pancreatitis pathology and improve prognosis for the treatment of AP in mice^11^. Thus, since dysfunctional autophagic flux is closely related to the early pancreatic inflammation, autophagosome formation is expected to become a new target for the treatment of pancreatitis.

MiR-155 has been widely studied in the immune system under conditions of normal and abnormal immune responses, inflammation, and cancer^5,31^. Taganov et al.^32^ found that LPS, TNF-α, and interleukin (IL)-1β stimulated human monocyte leukemia cells to upregulate some miRNAs, including miR-155. During the inflammatory response, miR-155 promotes inflammation and inhibits anti-inflammatory pathways by inhibiting the corresponding target proteins^6,33^. Previous studies showed that after LPS stimulation of alveolar macrophages, miR-155 increased autophagy formation by inhibiting TAB2^16^. TAB2 is an adaptor protein in the IL-1 signaling pathway, which could regulate autophagy by binding to the autophagy promoter Beclin-1^13^. When cells are treated with the autophagy inducer RAPA or PFT-α, TAB2 dissociates from beclin-1 and binds to TAK1, releasing beclin-1 to initiate autophagy. Based on these evidences, Criollo et al.^13^ proposed that TAB2 plays the role of “molecular switch” in autophagy. TAB2 is a target gene of miR-155 that is directly inhibited by this miRNA. Regarding pancreatitis, miR-155 expression levels were higher in patients with acute and chronic pancreatitis than in healthy controls, and thus, miR-155 levels should be associated with local and systemic inflammation^7,34^. Our study shows that AAV-9-mediated miR-155 delivery was efficient in the pancreas, and the expression of the target gene TAB2 was visibly regulated. Among AAV serotypes, AAV-9 showed more efficient pancreatic delivery in vivo, mediating long-term transgene expression with no immune response^35,36^. Thus, we compared the effect of miR-155 in the cerulein AP model with that in the cerulein plus LPS or L-arginine model. The results showed that miR-155 downregulation provides protection against AP damage by TAB2. The silencing of endogenous miR-155 could be an important mechanism to reduce impaired autophagy and decrease inflammation in AP (Fig. 7). Thus, regulating miR-155 may be a potential strategy for the treatment or prevention of pancreatitis.

## Supplementary information

Suppl figure 1

Suppl figure 2

Suppl figure 3

Suppl figure 4

Supplementary figure legends
