# Supplementary figures and images for: Inhibition of miR-155 reduces impaired autophagy and improves prognosis in an experimental pancreatitis mouse model
Source: Cell Death Dis. 2019 Apr 3;10(4):303. doi: 10.1038/s41419-019-1545-x (PMC6447551; doi:10.1038/s41419-019-1545-x)

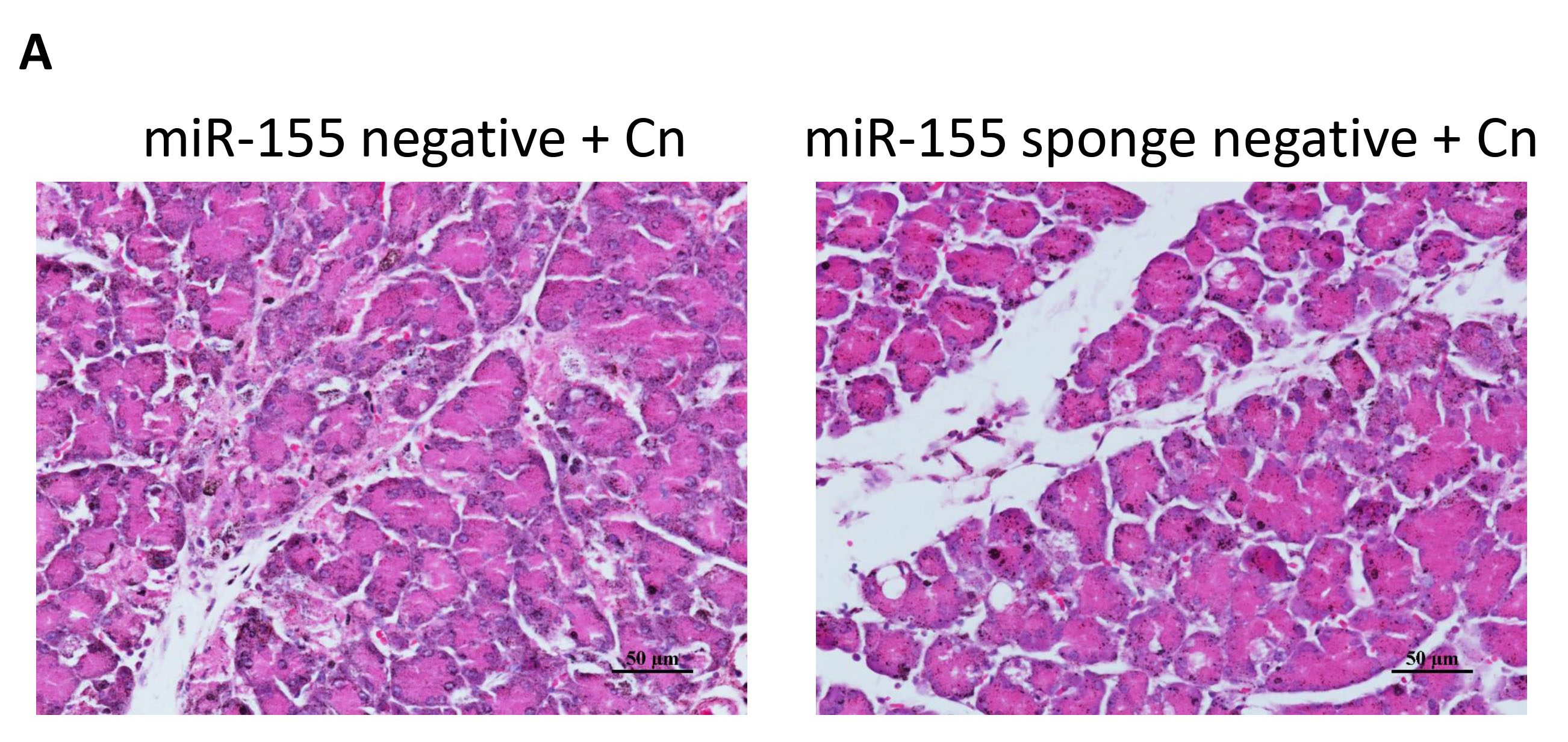

Supplement: Supplementary file 1 — Suppl figure 1 [file 41419_2019_1545_MOESM1_ESM.jpg]

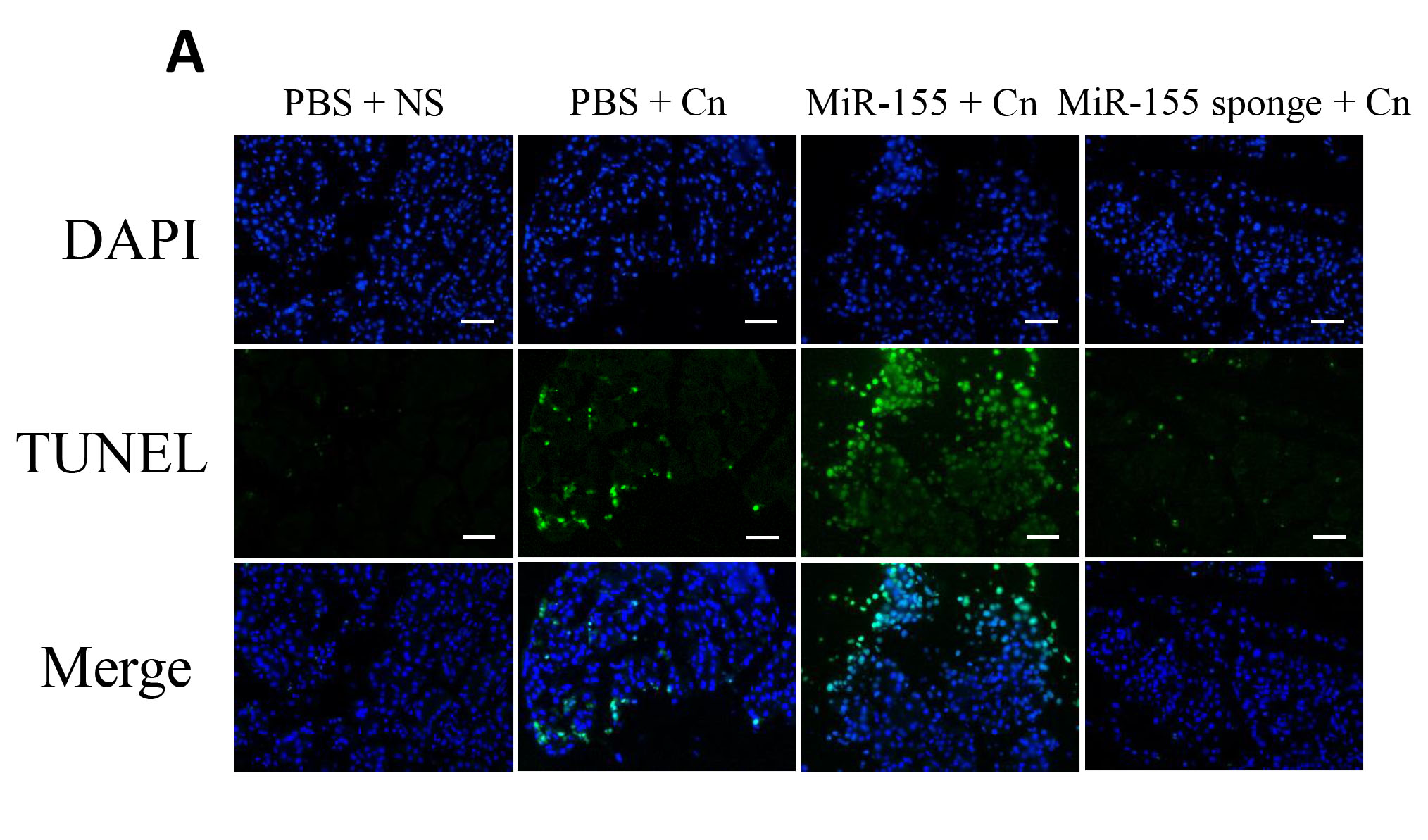

Supplement: Supplementary file 2 — Suppl figure 2 [file 41419_2019_1545_MOESM2_ESM.jpg]

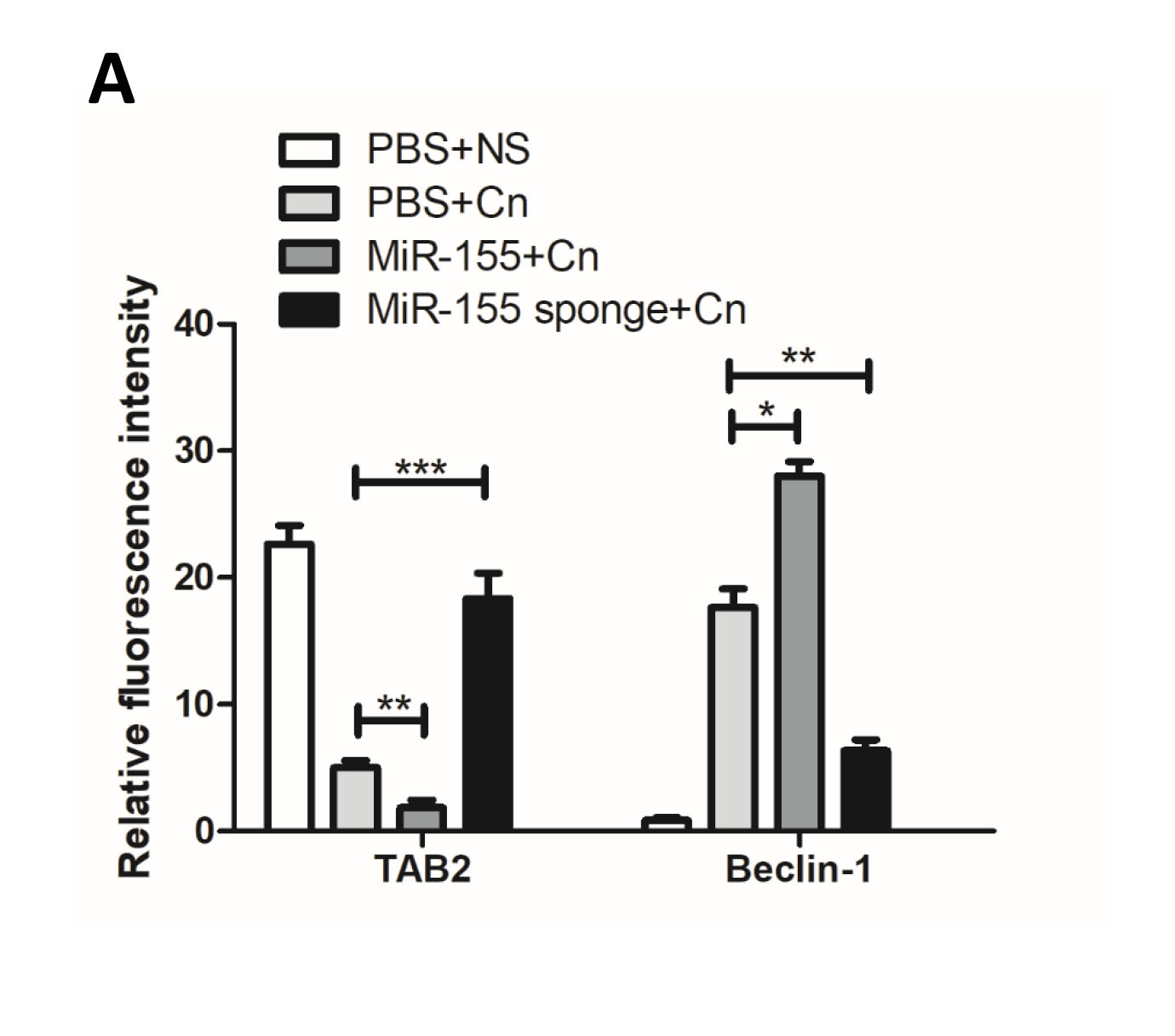

Supplement: Supplementary file 3 — Suppl figure 3 [file 41419_2019_1545_MOESM3_ESM.jpg]

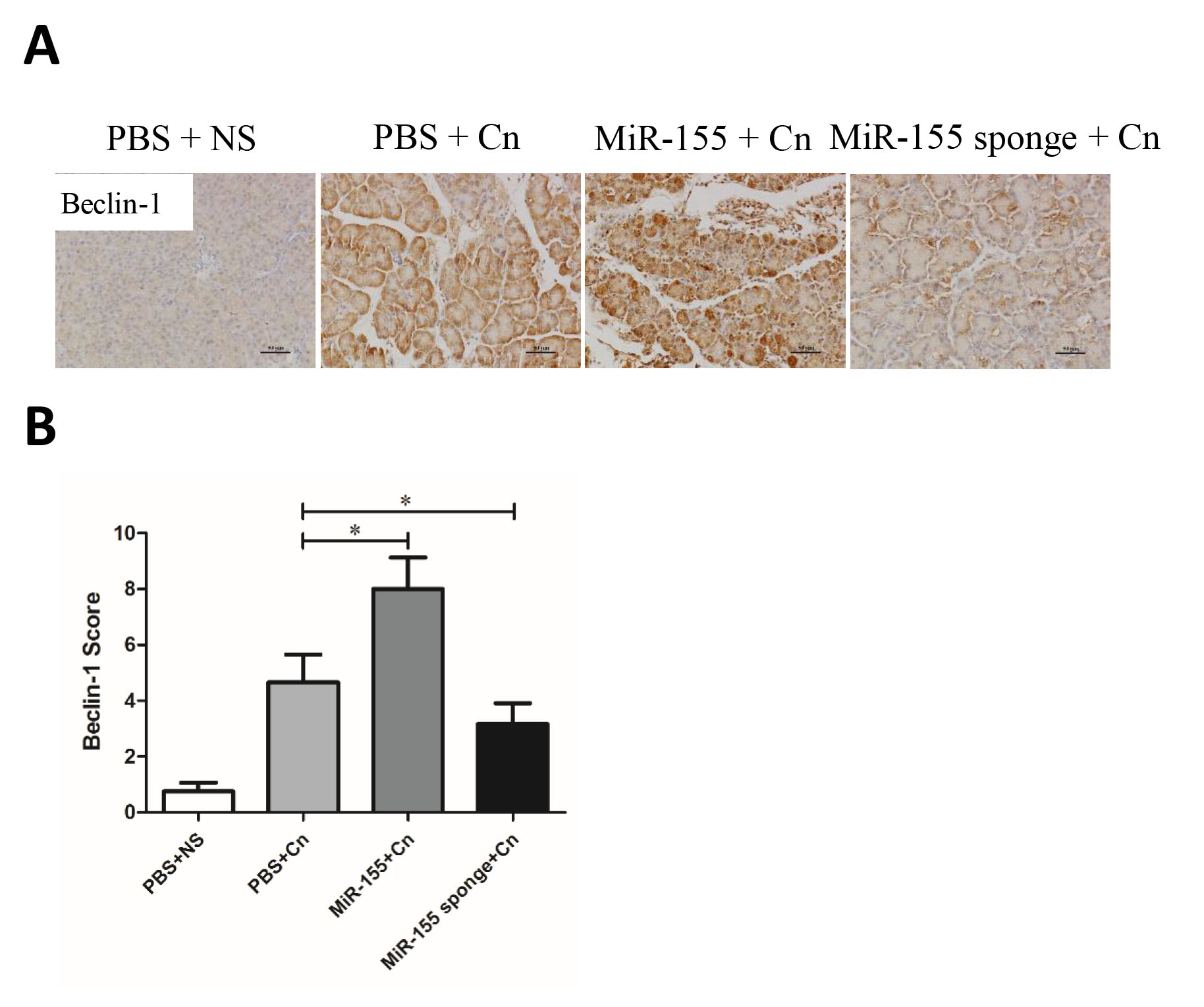

Supplement: Supplementary file 4 — Suppl figure 4 [file 41419_2019_1545_MOESM4_ESM.jpg]
