# Supplementary material for: Inhibition of miR-155 reduces impaired autophagy and improves prognosis in an experimental pancreatitis mouse model
Source: Cell Death Dis. 2019 Apr 3;10(4):303. doi: 10.1038/s41419-019-1545-x (PMC6447551; doi:10.1038/s41419-019-1545-x)
Supplement: Supplementary file 5 — Supplementary figure legends [file 41419_2019_1545_MOESM5_ESM.docx]

**Supplement Figure 1.** H&E staining of the pancreas from cerulein-treated mice administered the scrambled versions of miR-155 and miR-155 sponge.

**Supplement Figure 2.** Representative pictures of TUNEL staining to detect apoptosis in the AP group compared to the group treated with AAV-9-miR-155 or AAV-9-miR-155 sponge.

**Supplement Figure 3.** (A) Relative fluorescence intensity of TAB2 and beclin-1 in the pancreas (×200; green, TAB2; red, beclin-1; n = 5 per group, *P<0.05, **P<0.01, ***P<0.001).

**Supplement Figure 4.** (A) Immunohistochemical evaluation revealed the effect of miR-155 on autophagy based on beclin-1 expression levels in the pancreas (×200). (B) Immunohistochemical scores of beclin-1 staining (n = 6 per group, *P<0.05).
